# Occurrence and dietary exposure assessment of heavy metals in baby foods in the Kingdom of Saudi Arabia

**DOI:** 10.1002/fsn3.3485

**Published:** 2023-06-08

**Authors:** Najla S. Alharbi, Rawdah M. Akamsiei, Lama A. Almaiman, Mostafa A. AL‐Samti, Hamad S. Al‐Mutairi, Bandar S. Al‐owais, Majid M. Alkhalaf, Mohammed A. Bineid

**Affiliations:** ^1^ Executive Department of Monitoring and Risk Assessment, Food Sector Saudi Food and Drug Authority Riyadh Saudi Arabia; ^2^ Reference Laboratory for Food Chemistry Saudi Food and Drug Authority Riyadh Saudi Arabia; ^3^ National Nutrition Committee Saudi Food and Drug Authority Riyadh Saudi Arabia

**Keywords:** arsenic, cadmium, infant nutrition, lead, Saudi Arabia, toxic elements

## Abstract

Early childhood exposure to heavy metals like arsenic (As), cadmium (Cd), and lead (Pb) through baby foods unfolds many concerns about their toxic effects on growth and health. In this study, occurrence and dietary intake of As, Cd, and Pb in stage 1 infant formula (0–6 months), stage 2 infant formula (7–12 months), cereal‐based meals, and biscuits were estimated. First, the levels of As, Cd, and Pb were determined with ICP‐MS, followed by the calculation of estimated daily intake (EDI), target hazard quotient (THQ), and hazard index (HI) for As and Cd, and margin of exposure (MoE) for Pb. Mean levels of As, Cd, and Pb were the highest in cereal‐based meals and biscuits as 15.5–11.1, 5.18–8.76, and 35.2–53.8 μg/kg, respectively. Newborns to 6 months old infants were estimated to be the highest exposed population to Cd and Pb (0.08 and 0.36 μg/kg bw/day), while infants aged 7–12 months old were exposed the highest to As. Based on the THQ, HI, and MoE findings, the current exposure levels from the selected baby foods to As, Cd, and Pb pose low potential chronic risks to both infant age groups. This research provides a roadmap for future investigations in chemical contaminants often detected in baby foods consumed regularly by Saudi infants.

## Graphical abstract

Mean levels of As, Cd, and Pb were the highest in cereal‐based meals and biscuits. The current exposure levels from the selected baby foods to As, Cd, and Pb pose low potential chronic risks to both infants age groups.

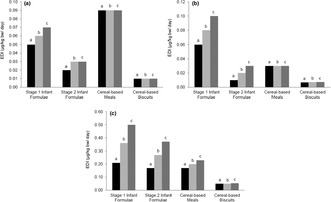

## 1 INTRODUCTION

Infant formulas and foods contribute to healthy infant nutrition, growth, and well‐being. It is especially essential in the cases of infants who depend majorly on formulas, do not get enough supplementary nutrients in their diet, or suffer of certain types of allergies (Su et al., 2020). Exclusive breastfeeding in the first 6 months is well addressed and recommended by the World Health Organization (WHO) and the Saudi Ministry of Health (MOH) to ensure the infant acquire the ideal nourishment at an early age (Ministry of Health Saudi Arabia, 2016; World Health Organization, 2016). Following the first 6 months, WHO suggests introducing complementary foods and beverages in the infant's diet alongside breast milk to offer the child the sufficient nutrients and proteins needed daily (Başaran, 2020). In Saudi Arabia, malnutrition markers of underweight, stunting, and wasting were investigated in 15,516 child under the age of 5 years (El Mouzan et al., 2010). According to the data, 8.2% of children younger than 5 years were diagnosed with underweight, while 12.7% and 13.7% were diagnosed with wasting and stunting, respectively (El Mouzan et al., 2010).

Many factors contribute to foodborne diseases occurrence and could be either biological such as bacteria and viruses, or chemicals including heavy metals, pesticides, or aflatoxins among many other toxins (AlFaris et al., 2020; World Health Organization, 2021). These toxins can be detected in most kinds of baby food items and can pose serious risks to the health of infants and young children (Khalifa & Ahmad, 2010). Chemical contaminants can be a key factor in developing many types of cancer, degenerative neurological diseases, inflamed infected systems, and reproductive toxicity (Onakpa et al., 2018; Rather et al., 2017). The presence of such agents in baby foods raise the concerns about their safety for consumption by a vulnerable age group and calls for regulatory actions to be determined (AlFaris et al., 2020).

Heavy metals in foods were investigated thoroughly in literature for decades. Recently, research interest investigating heavy metals in baby foods has risen vastly due to the sensitivity of the consumers and the risks these contaminants contribute to (Bargellini et al., 2018; Bundesinstitut für Risikobewertung, 2018; Khalifa & Ahmad, 2010; Su et al., 2020; Zhang et al., 2018). Generally, heavy metals migrate to food, including infant formula and processed baby food, products through different routes including soil, contaminated air and water systems, and inappropriate industrial practices (Jose & Ray, 2018; Kim et al., 2012). In addition, heavy metals can migrate to baby food chain from several anthropogenic activities such as improper storage conditions, poor farming practices, and disposal of chemical wastes (Jose & Ray, 2018). According to studies performed over the last decade, arsenic (As), cadmium (Cd), lead (Pb), and mercury (Hg) were the most detected heavy metals in infant formula, processed baby food in powder form, and ready‐to‐eat baby food items (Bargellini et al., 2018; Bundesinstitut für Risikobewertung, 2018; Khalifa & Ahmad, 2010; Su et al., 2020; Zhang et al., 2018). As, Cd, and Pb are naturally occurring contaminants that are widely distributed in air, soil, and water (Acharya, 2013; Martinez‐Finley et al., 2014; Zeece, 2020). The International Agency for Research on Cancer (IARC) has classified As and Cd as carcinogenic metals to humans (Group 1), while Pb was classified as possibly carcinogenic to humans (Group B2) (IARC, 2021).

In 2011, WHO released a technical document addressing the associated adverse effects of heavy metals on infants and children (World Health Organization, 2011). According to the WHO, repeated exposure of infants and young children to these heavy metals might cause many health complications including delayed physical and mental growth, poor immunological response, neurological impairment, and reproductive effects (Bundesinstitut für Risikobewertung, 2018; World Health Organization, 2011). Additionally, children exposure to heavy metal was observed to be associated with health complications in the respiratory and cardiovascular systems, as well as causing various types of cancer, such as childhood liver and kidney cancer (Capitão et al., 2022; Mbunga et al., 2022). It is essentially alarming when the child is exposed to such elements during pregnancy, infancy, and early childhood (WHO, 2021a). Arsenic chronic exposure may cause serious effects in children including liver cancer, while lead is mainly associated with neurological and developmental effects, due to its accumulation in bones and teeth (Mbunga et al., 2022; WHO, 2021a). Due to its accumulation in fat tissues, cadmium can be transmitted to fetus during pregnancy, as well as to infants through lactation (Mbunga et al., 2022). Similar to lead, cadmium may cause many adverse health effects on neurological system affecting the behavioral and mental development (Schoeters et al., 2006).

In 2011, JECFA withdrawn the provisional tolerable weekly intake (PTWI) for As for being no longer health protective, as the benchmark dose for a 0.5% (BMDL_0.5_) increased incidence of lung cancer was in the same range as the PTWI of 3 μg/kg bw/day (WHO, 2021c). For Cd, JECFA set a provisional tolerable monthly intake (PTMI) of 25 μg/kg bw/day based on negligible effects of daily exposure for adults and children (WHO, 2021d). For Pb, JECFA withdrawn the PTWI as it was associated with a decrease of at least 3 IQ points in children (WHO, 2021e). In 2010, EFSA established a BMDL_01_ of 0.5 μg/kg bw/day for Pb based on the developmental neurotoxicity in embryos, infants, and children younger than 7 years (European Food Safety Authority, 2010).

Considering the attributable risks associated with the exposure to heavy metals and the worldwide concerns about the contamination of infant formula and foods with heavy metals, it is essential to assess potential health risks of such contaminants in baby foods and monitor it on a regular basis. In 2020, occurrence data of heavy metals in baby foods in Saudi Arabia were obtained from the national food monitoring program (NFMP) executed by the Saudi Food and Drug Authority (SFDA). Based on such data, detailed health assessment was performed to evaluate noncarcinogenic risks. In this monitoring program, infant formula and cereal‐based baby foods contaminated with As, Cd, and Pb have been selected for the assessment of dietary exposure and chronic noncarcinogenic risks associated with consumption for infants in the age group of 0–12 months old. Due to the inadequacy of thorough risk assessment reports of heavy metals in baby foods in Saudi Arabia, as well as the lack of maximum limits for certain types of heavy metals in baby foods, such as cereal meals and biscuits, this study was performed. Additionally, this study aims to assess the applicability of suggested maximum limits for heavy metals on such food items in Saudi Arabia. To the best of our knowledge, this is the first study that estimates the full risk assessment of Saudi infants to heavy metals in baby foods. Such results will provide basic data for food scientists and regulators in Saudi Arabia and in Middle East, as well as other importing countries of baby foods.

## 2 METHODOLOGY

### 2.1 Sample collection

Data of 111 samples of commercially available food products for infants (newborns to 12 months) were collected and analyzed for heavy metals contamination during the 2020 NFMP executed by the SFDA. Toxic elements targeted in this study for baby food products are As, Cd, and Pb. Samples were collected throughout the year 2020 from pharmacies and main markets of three regions (Riyadh, Jeddah, and Dammam), Kingdom of Saudi Arabia. The baby food products under analysis were 39 samples of stage 1 infant formula (0–6 months), 22 samples of stage 2 (7–12 months), 33 sample of processed cereal‐based meals, and 17 samples of biscuits. Since most baby foods retailed in Saudi Arabia are imported, samples selected in this study consisted of 21 and 90 samples of domestic and imported products, respectively. These products were selected based on market share data of baby food brands commonly retailed in Saudi Arabia throughout the year 2020. The collected samples were preserved at room temperature and sent immediately to corresponding SFDA laboratories in the three regions selected.

### 2.2 Analytical procedure

#### 2.2.1 Reagents and chemicals

All reagents used were of ultra pure grade. High purity nitric acid (65.0%) was provided by Romil Pure Chemistry. The multielement standard solution (containing: As, Cd, and Pb at 10.0 mg/L) and the internal standard solution (100 mg/mL) were obtained from Agilent.

#### 2.2.2 Sample preparation and extraction

For the preparation of a test sample, 200 g should be available from the laboratory sample. The sample was homogenized by a blender until each baby food product became a fine powder using a Titanium homogenizer (Knief Mill, GM 200; Retsch). All materials used were sufficiently clean to avoid any contamination. A homogenized sample (0.50 g) was weighed into quarts vessels with 5 mL ultrapure nitric acid (65.0% v/v) and the reaction was accelerated in a pressure‐ and heat‐controlled Ultraclave microwave digestion system (Milestone ultraCLAVE) programmed to ramp temperature from 60.0 to 200°C over a 30‐min cycle, and at 120 bar pressure. Samples were then diluted in centrifuge tubes to 50 mL (a total dilution factor of 100) with deionized water (high purity Milli‐Q grade 1, Millipore). An aliquot of 6 mL of the sample was pipetted into disposable ICP‐MS vials without filtration (European Standards, 2023b).

#### 2.2.3 Analysis condition

An inductively coupled plasma mass spectrometry quadrupole mass spectrometer with collision cell technology (7700 ICP‐MS Agilent) is used for metal analysis. the radio frequency power for the ICP was set at 1500 W, and the plasma gas flow was 15.0 L/min, while the auxiliary (makeup) gas flow was 1.00 L/min. Helium gas was used in the collision cell with a flow rate 4.50 mL/min. Before injecting the samples, sensitivity, oxide ratio, and doubly charged ratio specifications were checked to minimize interferences. There are two choices of mode to determine metals analysis (no gas/helium) mode, all results were approved in helium mode. Recommended atomic masses for the elements under study are As 75, Cd 111, and Pb 208 amu. ICP‐MS method was applied and validated for the analysis of food including baby food samples according to the European standards (CSN EN 15763, BS EN 13804:2013) (European Standards, 2023a, 2023b).

#### 2.2.4 Quality control

The validation was performed according to the internal validation procedure developed according to Eurachem guidelines (Eurachem, 2023). To perform recovery studies in baby food matrix, infant milk powder samples were fortified at three levels of concentrations 10, 50, and 100 μg/kg in eight replicates. The accepted recovery was set between 70.0% and 120%, while relative standard deviations (RSDs) of repeated measurements were set at <20.0% (Table 1). Evaluation of selectivity was performed using blank samples with no response of all analytes (As, Cd, and Pb). In addition, accuracy was evaluated based on the recovery rate, which should be between 70.0% and 120% as shown in Table 1. Furthermore, repeatability and reproducibility are assessed based on relative standard deviation (%RSD) which should not exceed (±20.0). The linearity range described by a correlation coefficient (*R* ^2^) was confirmed to be >1.00. Additional quality control techniques used were solvent blank, fresh calibration standards, and spiked samples. Certified reference material was prepared and analyzed with each analysis batch to ensure the reliability and accuracy of the results.

**TABLE 1:** Method validation data.

Analyte	Baby food matrix					
	LOD^a^ (μg/L)	LOQ^b^ (μg/L)	LDR^c^ (μg/L)	*R* ^2^	Recovery %	RSD^d^ %
As	0.70	2.40	0–100	1.00	116	6.42
Cd	0.90	3.00	0–100	1.00	106	3.28
Pb	6.90	23	0–100	1.00	108	3.83

^a^ Limit of detection.

^b^ Limit of quantification.

^c^ Linear dynamic range.

^d^ Relative standard deviation (*n* = 8).

### 2.3 Risk assessment

#### 2.3.1 Consumption data

Considering the globally unified consumption of formula milk by infants, and due to limited data of consumption of formula milk in Saudi Arabia and Middle East in general, Estimated daily consumption (EDC) of baby food was calculated based on scoops and portion sizes recommended daily on the packaging as shown in Equation (1) next:

(1) $$\mathrm{EDC}\;\left(g/\mathrm{day}\right)=\left(\mathrm{no}.\;\text{scopes}\times \text{scope size}\;\left(g\right)\right)\times \mathrm{no}.\;\text{feeds}\;\mathrm{per}\;\mathrm{day}$$

For infants aged 0–6 months and consuming stage 1 formula milk only, the recommended number of feeds per day ranged between 5 and 6 times/day. The estimated average daily consumption was 112 g/day, as recommended on the labels of the products (five scopes each one of 4.50 g/formula bottle). For infants aged 7–12 months, the recommended number of feeds per day ranged between 3 and 5 times/day. The estimated average daily consumption was calculated as recommended on the label of stage 2 infant formula was 129 g/day (seven scopes each one of 4.60 g/ formula bottle). For the other cereal‐based food products, the recommended daily number of cereal‐based meals was one meal per day and was calculated as recommended on the label (50 g/meal), while the recommended daily number of biscuits were one stick per day (the stick weigh 8 g as described on the packaging).

#### 2.3.2 Estimated dietary intake (EDI)

EDI values were estimated using mean daily consumption (CONS) in g/day, body weight (BW) of Saudi infants in kg (0–6 months and 7–12 months infants averaged 5.45 kg and 8.90 kg, respectively [Ministry of Health, 2021a]), and mean contaminant content (C) in μg/kg. Equation (2) represents the exposure of infants to toxic elements through selected baby foods.

(2) $$\mathrm{EDI}\;\left(\mathrm{\mu g}/\mathrm{kg}\;\mathrm{bw}/\mathrm{day}\right)=\left(\left(C\;\left(\mathrm{\mu g}\right)\times \text{CONS}\;\left(g/\mathrm{day}\right)\right)/1000\;\left(g\right)\right)/\mathrm{BW}\;\left(\mathrm{kg}\right)$$

#### 2.3.3 Hazard characterization

##### Target hazard quotient

Target hazard quotient (THQ) represents the probable chronic risk from exposure to toxic elements present in food (Sonomdagva et al., 2019; US EPA, 2021a). This value was applied only to estimate the noncarcinogenic effects of As and Cd (US EPA, 2021a). Equation (3) below summarizes the estimation of THQ of As and Cd in selected baby foods. A THQ value of ≤1.00 indicates that the risk of developing noncarcinogenic effects is unlikely (US EPA, 2021a). However, a THQ value >1.00 indicates increased health risks of a given contaminant (US EPA, 2021a).

(3) THQ=EDI/RfD

Reference dose (RfD) describes an assessment of oral daily intake that is likely to be without deleterious effects during a lifetime for the general population (Hurt et al., 2010). According to the Integrated Risk Information System (IRIS) of the US Environmental Protection Agency (USEPA), RfD values of As and Cd were estimated as 0.30 and 1.00 μg/kg bw/day, respectively (US EPA & IRIS, 2021).

##### Hazard index

Hazard index (HI) refers to the cumulative noncarcinogenic risks caused by multiple toxic elements that share combined adverse effects through food ingestion (US EPA, 2021a). HI was assessed using the sum of THQ values estimated for all the targeted contaminants, which was represented in Equation (4) below (US EPA, 2021a). An HI ≤1.00 indicates no risk for the human health (US EPA, 2021a).

(4) HI=∑THQ

##### Margin of exposure

Margin of exposure (MOE) approach was adopted to evaluate the risks related to human health associated with Pb in baby foods. This was carried out due to lack of appropriate RfD and HBGVs for Pb (European Food Safety Authority, 2010; US EPA, 2021b). MOE is the ratio of a benchmark dose lower limit (BMDL_01_) and the human EDI to evaluate the cancer risk upon chronic exposure, as illustrated in Equation (5) below (Trier et al., 2010). According to EFSA, a MOE ≥10.0 is considered as safe for consumption during a lifetime (for both adults and children) with low risks of developing noncarcinogenic effects, such as developmental neurotoxicity in infants and children (European Food Safety Authority, 2010).

(5) MOE=BMDL/EDI

### 2.4 Statistical analysis

Statistical analysis was performed using Microsoft Excel 2016. Mean and standard deviation values for heavy metals content in the selected samples were determined. Statistical treatment of left‐censored data was based on the standard approach by Codex Committee on Contaminants in Foods (CL 2021/78‐CF) (CCCF, 2021). The method include using upper bound (UB), middle bound (MB), and lower bound (LB) and is commonly used to assess the content of chemical contaminants found in food. For LB approach, results reported to be <LOQ or <LOD should be treated as zero. For MB approach, results reported to be <LOQ or <LOD should be treated as 1/2 LOQ or 1/2 LOD. For UB approach, left‐censored data <LOQ or <LOD should be treated as LOQ or LOD value, respectively. Differences and homogeneity of variance in mean content of heavy metal among infant formula samples for both stages 1 and 2 were obtained by Levene's test. In order to validate whether significant differences (*p* < 0.05) between various types of baby foods were present, nonparametric Kruskal–Wallis and the Mann–Whitney tests were applied. The tests were select in view of the fact that the current data did not follow a normal distribution. All tests were performed using IBM SPSS statistics for windows Version 21 (IBM Corp.). The *p* ≤ .05 was considered statistically significant.

## 3 RESULTS

### 3.1 Occurrence data

**FIGURE 1:**
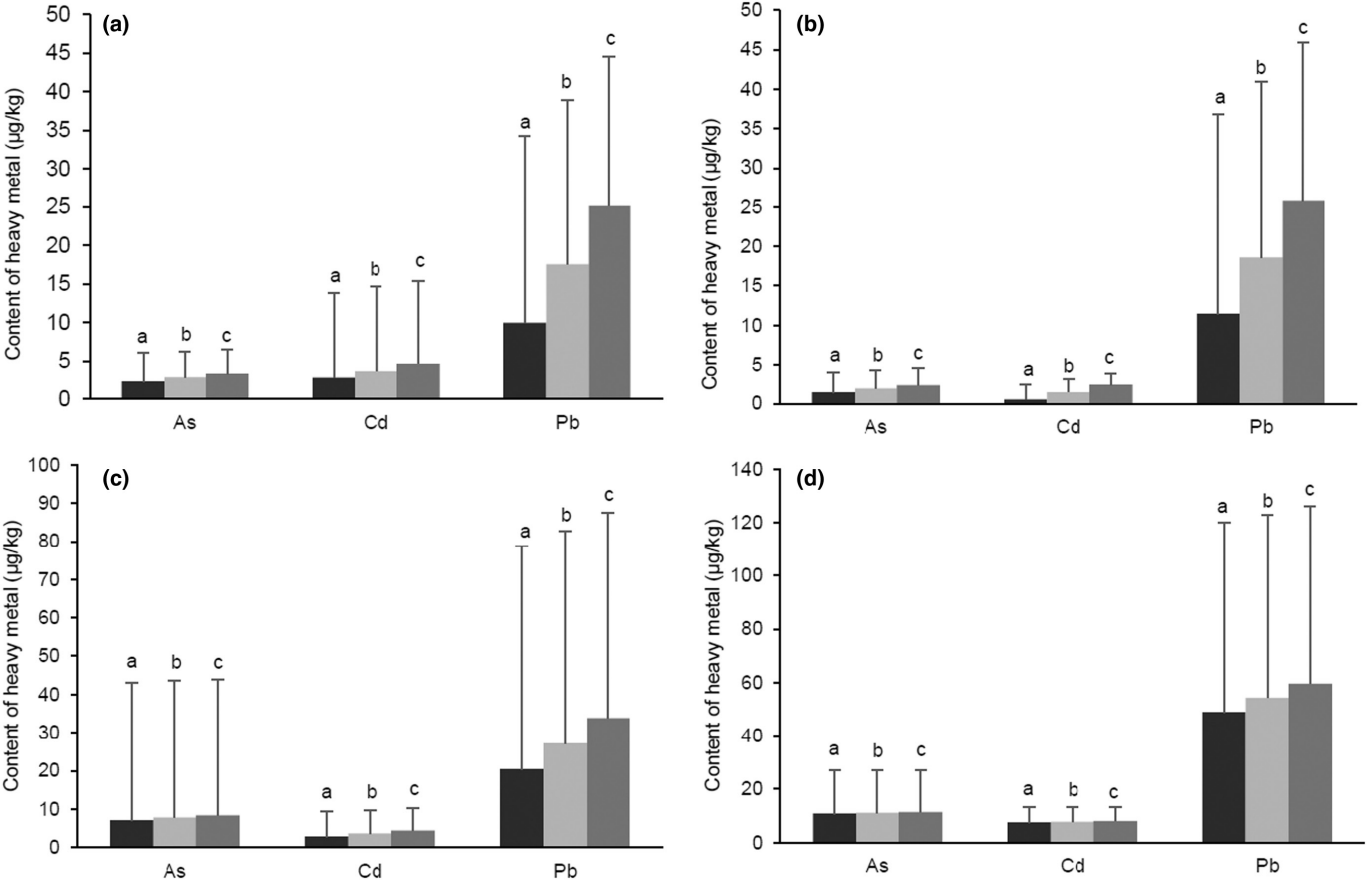
Mean contents of toxic elements As, Cd, and Pb detected in baby foods based on lower bound (LB), middle bound (MB), and upper bound (UB) approaches. (a) Contents of contaminants detected in stage 1 infant formula. (b) Contents of contaminants detected in stage 2 infant formula. (c) Contents of contaminants detected in cereal‐based meals. (d) Contents of contaminants detected in cereal‐based biscuits. Data are presented using *t* test and Kruskal–Wallis test as mean and standard deviation (SD) (*n* = 4). a = lower bound (LB) mean content of contaminant, b = middle bound (MB) mean content of contaminant, c = upper bound (UB) mean content of contaminant. *p ≤ .05 was considered statistically significant.

In this study, As, Cd, and Pb were determined in all selected samples (Figure 1). For stage 1 infant formula, the levels of toxic elements (MB) were ranged from 0.35 to 15.0 μg As/kg, 0.50 to 60.0 μg Cd/kg, and 3.80 to 119 μg Pb/kg. For stage 2 infant formula, the toxic elements contents (MB) ranged from 0.35 to 8.20 μg As/kg, 0.50 to 7.30 μg Cd/kg, and 3.80 to 104 μg Pb/kg. For cereal‐based meals, the contents of toxic elements (MB) ranged from 0.35 to 179 μg As/kg, 0.50 to 28.0 μg Cd/kg, and 3.80 to 255 μg Pb/kg. For biscuits, the toxic elements contents (MB) ranged from 0.35 to 53.0 μg As/kg, 0.50 to 21.9 μg Cd/kg, and 3.80 to 223 μg Pb/kg. There were no significant statistical differences among all contaminants present in stage 1 and 2 infant formula samples (As *p* [MB] .258 > .05, Cd *p* [MB] .335 > .05, Pb *p* [MB] .858 > .05). No significant differences were identified among As and Pb levels present in all baby food groups (As *p* [MB] .120 > .05, Pb *p* [MB] .091 > .05). However, the Kruskal–Wallis test showed significant differences in Cd levels between all baby food groups (Cd *p* [MB] .000 < .05). Mann–Whitney test was used to validate the significant differences in Cd contents between all baby food types, and it showed significant differences between infant formula and cereal‐based meals (*p* [MB] .000 < .05), infant formula and biscuits (*p* [MB] .000 < .05), cereal‐based meals and biscuits (*p* [MB] .007 < .05).

The lowest mean concentrations of the contaminants were determined mainly in stage 1 infant formula, while the highest mean concentrations were observed in biscuits. The highest mean content of As was observed in cereal‐based meals and biscuits (15.5 ± 35.8 μg As/kg in cereal‐based meals, 11.1 ± 15.9 μg As/kg in biscuits). The highest mean content of Cd was observed in cereal‐based biscuits (8.76 ± 5.56 μg Cd/kg) followed by cereal‐based meals (5.18 ± 6.16 μg Cd/kg) and stage 2 infant formula (1.47 ± 1.63 μg Cd/kg). Pb mean contents were found the highest in biscuits (53.8 ± 68.6 μg Pb/kg), followed by cereal‐based meals (35.2 ± 55.7 μg Pb/kg).

### 3.2 Risk assessment: daily intake estimation, target hazard quotient, hazard index, and margin of exposure

**FIGURE 2:**
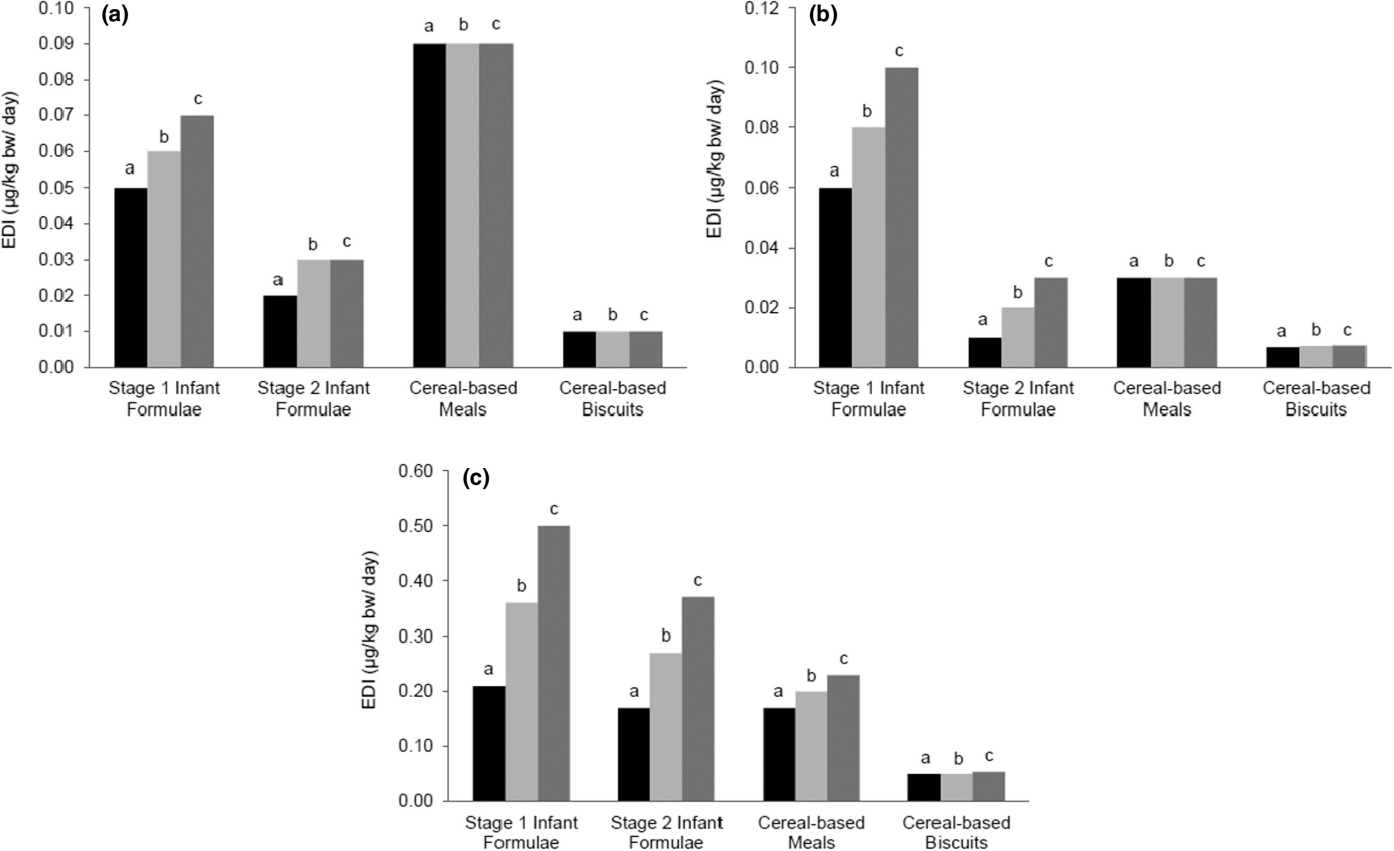
Lower bound (LB), middle bound (MB), and upper bound (UB) of the estimated daily intake (EDI) of As, Cd, and Pb detected in selected baby foods. EDI is evaluated in baby foods for of (a) arsenic (As), (b) cadmium (Cd), (c) lead (Pb). All data are represented using Microsoft Excel 2016. a = lower bound (LB) mean content of contaminant, b = middle bound (MB) mean content of contaminant, c = upper bound (UB) mean content of contaminant.

Dietary exposure to toxic elements investigated in this study was estimated for two age groups: 0–6 and 7–12 months. For the first group (0–6 months), exposure assessment was based on an infant diet consisted of commercial formula only. For the second group (7–12 months), the assessment was based on a diet of commercial infant formula stage 2 and cereal‐based meals and biscuits only (for 7–12 months). Total intakes of heavy metals for both age groups based on LB, MB, and UB approaches were presented in Figure 2 and Table 2. As and Cd were assessed for noncarcinogenic chronic risks associated with the consumption of baby foods using target hazard quotient (THQ) and hazard index (HI) based on LB, MB, and UB estimates (Table 3). LB, MB, and UB estimates of MOE of Pb in selected baby foods based on the EDI of each food group and Pb BMDL_01_ of neurodevelopmental toxicity were estimated as shown in Table 4.

**TABLE 2:** The estimated dietary intake (EDI) of As, Cd, and Pb for age groups: 0–6 months and 7–12 months based on the recommended serving size on the label of each product. The unit is μg/kg bw/day.

Heavy metal	Total EDI of infant formula (0–6 months)	Total EDI of baby foods (7–12 months)
As		
LB	0.05	0.04
MB	0.06	0.05
UB	0.07	0.06
Cd		
LB	0.06	0.01
MB	0.08	0.02
UB	0.10	0.02
Pb		
LB	0.21	0.10
MB	0.36	0.17
UB	0.50	0.21

**TABLE 3:** Target hazard quotient (THQ) and hazard index (HI) of As and Cd in selected baby foods.

Food group	Heavy metal	THQ			HI		
		LB	MB	UB	LB	MB	UB
Stage 1 infant formula only (0–6 months)	As	0.17	0.20	0.23	0.23	0.28	0.33
	Cd	0.06	0.08	0.10			
Baby foods (7–12 months)	As	0.13	0.17	0.20	0.14	0.19	0.22
	Cd	0.01	0.02	0.02			

**TABLE 4:** Margin of exposure (MOE) of Pb in selected baby foods.

Food group	MOE		
	LB	MB	UB
Stage 1 infant formula only (0–6 months)	2.38	1.67	1.00
Baby foods (6–12 months)	5.00	2.94	2.38

Intake estimation of As for 0–6 months was below the daily RfD set by USEPA for As, which was 0.30 μg/kg bw/day based on cardiovascular effects, and As contribution to the total intake was 19.5%. The intake of commercial infant formula (0–6 months) ranged from 0.05 to 0.07 μg/kg bw/day (LB‐UB), as shown in Figure 2. Similarly, the total intake of the second group (7–12 months) was below the RfD and ranged from 0.04 to 0.06 μg/kg bw/day (LB‐UB) (Figure 2). It has been observed that the highest contribution to As exposure was by the consumption of cereal‐based meals and accounted for 29.1% of total intake. This was followed by stage 2 formula milk that contributed to approximately 9.18% of total intake, while biscuits contributed to 3.32%. THQ values of As were all <1.00 as shown in Table 3.

Intake estimation of Cd for 0–6 months was below the daily PTWI set by EFSA for Cd as 2.50 μg/kg bw/week (corresponding to PTDI of 0.36 μg/kg bw/day) based on cardiovascular effects, and the mean contribution of infant formula was 21.4% (Figure 2). The intake of Cd from commercial infant formula (0–6 months) ranged from 0.06 to 0.10 μg/kg bw/day (LB‐UB). Similarly, the total intake of Cd for the second group (7–12 months) from various baby foods was below the PTDI and ranged from 0.01 to 0.02 μg/kg bw/day (LB‐UB) as shown in Figure 2. The highest contribution to Cd exposure for 7–12 months diet was by the consumption of cereal‐based meals and stage 2 formula milk and accounted for 8.08% and 5.89%, respectively, while biscuits contributed to 2.19% of total intake (Figure 2). THQ of Cd and HI values based on LB, MB, and UB levels were all <1.00, as shown in Table 3.

Intake estimation of Pb by 0–6 months old infants based on infant formula only was 0.21–0.50 μg/kg bw/day (LB‐UB). For embryos, infants, and children ≤7 years, a benchmark dose (BMDL_01_) of Pb was determined as 0.50 μg/kg bw/day based on the observed neurodevelopmental toxicity and decreased IQ (European Food Safety Authority, 2010). Hence, the estimated Pb EDI for 0–6 months age group was ≤ BMDL_01_. On the other hand, the estimated EDI for the second group (7–12 months) was below the BMDL and ranged from 0.10 to 0.21 μg/kg bw/day (LB‐UB). Figure 2 and Table 2 illustrate the estimation of Pb intake for all groups under investigation.

Due to lack of a health‐based benchmark of lead in food, and since it is inappropriate to derive one due to the associated adverse effects of lead, applying the margin of exposure (MOE) is crucial to determine the health risks of lead on embryos, infants, and children (European Food Safety Authority, 2010; US EPA, 2021b). According to EFSA, a MOE of ≥10.0 indicates low risks associated with effects and IQ and neurodevelopmental toxicity (European Food Safety Authority, 2010). At lower MOEs, but greater than 1.00, the risk is likely to be low, but not such that it could be dismissed as of no potential concern (European Food Safety Authority, 2010). Table 4 demonstrates LB, MB, and UB estimates of MOE of lead in baby foods based on the EDI of each food group and lead BMDL_01_ of neurodevelopmental toxicity.

## 4 DISCUSSION

### 4.1 Occurrence data

As levels in all baby food samples in this study were lower than the levels reported previously by Al‐Khalifa and Ahmad in 2010 (Table 5) (Khalifa & Ahmad, 2010). Considering such evidence, our findings may indicate the development of Saudi regulatory and monitoring systems on baby foods retailed in Saudi Arabia between 2010 and 2020, in addition to the improvement of manufactural practices since 2010. Similarly, our results of As in a number of baby food samples tested were lower than recent reports of Turkey, Poland, Nigeria, Spain, and Australia (Table 5) (Başaran, 2022; Gu et al., 2020; Su et al., 2020). However, As contents in infant formula stage 1 and cereal‐based meals in this study were higher than observed in similar studies of China and Poland (Mania et al., 2015; Su et al., 2020). For the last decade, rice has been a debatable topic of concern, particularly on infants and small children (U.S. Food and Drug Administration, 2016). According to WHO, As is naturally present in soil, water, and cereal crops (WHO, 2021b). Rice, in particular, tend to contain high levels of As due to its vast occurrence, among many other toxic elements, in polluted groundwater used in paddy fields (Chen et al., 2017; WHO, 2021b). Previous studies suggested that rice plants highly accumulate As compared to other grains such as wheat and barley (Rahman & Hasegawa, 2011; Williams et al., 2007). Many baby food products including cereals, puffs, and biscuits are made mainly of rice because of its nutrients availability, dull flavor, and low allergenicity (Gu et al., 2020; Signes‐Pastor et al., 2016). Preparing commercial cereal‐based meals with As‐contaminated cooking water could contribute to higher As retention and exposure (Bae et al., 2002; Kumarathilaka et al., 2019). Such fact is alarming due to infants high susceptibility to As adverse effects compared to adults and older children (U.S. Food and Drug Administration, 2016).

**TABLE 5:** Contents of toxic elements As, Cd, and Pb detected in different baby foods as reported in other studies.

Food item	Year	Country	Mean content (μg/kg)			Reference
			As	Cd	Pb	
Stage 1 infant formula	2010	Saudi Arabia	89.0	7.00	18.0	Khalifa and Ahmad (2010)
Stage 2 infant formula			80.0	2.00	5.00	
Cereal‐based meals			170	18.0	23.0	
Biscuits			710	7.00	37.0	
Infant formula	2015	Poland	6.70	1.20	5.10	Mania et al. (2015)
Mixed cereals meals			10.2	3.50	6.40	
Biscuits			8.70	6.60	12.6	
Baby foods	2016	Serbia	<LOD	<LOD	<LOD	Škrbić et al. (2017)
		Spain	40.0	<LOD	9.00	
Infant formula	2018	Italy	–^a^	10.8	20.6	Bargellini et al. (2018)
Infant formula	2018	Germany	–^a^	3.00	10.0	Bundesinstitut für Risikobewertung (2018)
Cereal‐based meals				19.0	8.00	
Biscuits				13.0	7.00	
Stage 1 infant formula	2019	Egypt	–^a^	8.00	N.D.	Ghuniem et al. (2020)
Stage 2 infant formula			–^a^	9.00	N.D.	
Infant formula	2019	United States	–^a^	0.00	0.00	Gardener et al. (2019)
Cereal‐based products			–^a^	2.76	0.00	
Rice cereal	2020	Australia	134	–^a^	–^a^	Gu et al. (2020)
Infant formula	2020	Nigeria	330	–^a^	–^a^	Igweze et al. (2020)
Stage 1 infant formula	2020	China	3.00	0.88	1.61	Su et al. (2020)
Stage 2 infant formula			3.57	1.01	2.19	
Stage 1 infant formula	2021	Poland	–^a^	–^a^	0.00	Maruszewska et al. (2021)
Stage 2 infant formula					0.01	
Infant formula	2021	Turkey	21.0	2.00	25.0	Başaran (2022)

Abbreviations: LOD, limit of detection; N.D., not detected.

^a^ Not targeted or tested in corresponding study.

Current levels of Cd tested in baby foods were consistent with previous reports (Table 5) in Saudi Arabia, Turkey, Germany, Poland, and China (Başaran, 2022; Bundesinstitut für Risikobewertung, 2018; Khalifa & Ahmad, 2010; Mania et al., 2015; Su et al., 2020). Cd contents in infant formula analyzed in this study were lower than the contents observed in Italy and Egypt (Bargellini et al., 2018; Ghuniem et al., 2020). In contrast, our results were higher than Cd contents reported in various baby foods in United States, Serbia, and Spain (Gardener et al., 2019; Škrbić et al., 2017). Although low contents of Cd and As were detected in the analyzed baby food samples, long‐term exposure to such toxic and carcinogenic metals with a diet based solely on formula and commercial baby foods is a matter of concern, and preventive measures should be considered. Similar to As, Cd can accumulate in the food chain including rice and other cereals and grains used for infant foods due to both natural and human‐induced actions such as excessive use of pesticides and fertilizers (de Paiva et al., 2019; Medfu Tarekegn et al., 2020). Moreover, heavy metals cannot be degraded or eliminated from foods completely, and can accumulate in the body for a long time; thus, posing serious risks on consumers, especially infants and young children (Schaefer et al., 2020).

Previous literature of Saudi Arabia, Italy, and Turkey (Table 5) has demonstrated the presence of Pb levels in baby food samples exceeding the EU and SFDA limits (Bargellini et al., 2018; Başaran, 2022; Khalifa & Ahmad, 2010). Nevertheless, our findings were higher compared to these reports and to Pb levels analyzed in Germany, United States, China, Spain, Poland, and Eygpt (Bae et al., 2002; Bundesinstitut für Risikobewertung, 2018; Chen et al., 2017; Gardener et al., 2019; Ghuniem et al., 2020; Kumarathilaka et al., 2019; Mania et al., 2015; Maruszewska et al., 2021; Rahman & Hasegawa, 2011; Signes‐Pastor et al., 2016; Škrbić et al., 2017; Su et al., 2020; U.S. Food and Drug Administration, 2016; WHO, 2021b; Williams et al., 2007). Even though there were no significant differences in the contents of Pb in infant formula between 2010 and 2020 in Saudi Arabia, Pb levels determined in the 2020 NFMP were lower than the levels reported in Saudi Arabia in 2010 of stage 1 infant formula (Khalifa & Ahmad, 2010). While on the other hand, Pb contents detected in stage 2 infant formula were higher than the results presented in Al‐Khalifa and Ahmad (2010).

According to our outcomes, cereal‐based products were identified as common components associated with elevated Pb contents. Similarly to As and Cd, Pb is distributed widely in food products naturally and by anthropogenic acts (WHO, 2021a). According to WHO, infants and young children are extremely susceptible to Pb‐induced neurotoxicity and decreased IQ levels even at low exposure levels due to its ability of accumulating in the body on the longer term (US EPA, 2021c; WHO, 2021a). Considering infants sensitive age, direct contact of hand and mouth, and the consumption of a commercial‐baby‐foods‐only diet, the associated risks with Pb exposure may increase highly (WHO, 2021a). In addition, tap water or drinking water used for preparing formula bottles may contain heavy metals content among which As, Cd, and Pb; therefore, increasing the content an infant may be exposed to per day (Elaridi et al., 2021; US EPA, 2021c).

The current contents of heavy metals in this study could indicate the contribution of other external factors, for instance manufactural practices including the processing of baby foods, production, and the use of food‐contact materials (Fiamegos et al., 2016; Srivastava et al., 2017). Machines and instruments used in such acts might play a significant part in transporting heavy metals to baby food products (Fiamegos et al., 2016; Srivastava et al., 2017). Moreover, heavy metals may occur in packaging materials and migrate to infant formula, meals, and biscuits under poor storage conditions such as temperature, light, and moisture (Fiamegos et al., 2016; Srivastava et al., 2017). Sample handling and extraction could participate further in heavy metals contamination as observed in this study and others, in addition to chemicals and analytical techniques and instruments used in such processes (Almutairi et al., 2021).

### 4.2 Risk assessment: daily intake estimation, target hazard quotient, hazard index, and margin of exposure

The intake values were assumed as results of dry (as sold), undiluted infant formula and cereal‐based meals, without considering the use of water in the process. Other sources of exposure to heavy metals might contribute to increased exposure, for example, drinking water, ready‐to‐eat purees in jars, gruels, baby juices, artificial toys, feeding bottles, pacifiers, and teethers (Aboel Dahab et al., 2016; Koester et al., 2021; Mania et al., 2015). Compared to adults, infants and toddlers tend to eat larger portions of food with smaller body weight ratio; exposing such sensitive group to higher dietary risks associated with toxic elements under investigation (Gu et al., 2020; Mielech et al., 2021). Due to their natural occurrence in nature and food, heavy metals cannot be completely excluded from foods and drinking water (Pettoello‐Mantovani et al., 2021). Reducing the consumption of commercial cereal‐based meals and snacks by preparing homemade purees and gruels of fruits and vegetables may lower infants and young children exposure to heavy metals (Gardener et al., 2019; Pettoello‐Mantovani et al., 2021; U.S. Food and Drug Administration, 2016).

THQ values of As were all <1.00 as shown in Table 3, indicating the low chronic risks associated with a diet of commercial baby foods only. Such outcomes were consistent with recent literature reported in Poland, Spain, China, and Australia (Gu et al., 2020; Mania et al., 2015; Škrbić et al., 2017; Su et al., 2020). However, the previous outcomes does not essentially mean that As is not harmful in early childhood, particularly on infants aged less than 6 months old. Infants at this age are the most sensitive and susceptible to toxic effects of As, especially if their diet is limited on commercial formula only. As presence even in small quantities is concerning on the development of infants and young children (de Mendonça Pereira et al., 2020). Toxic impacts on cognitive development, intelligence, and renal and pulmonary functions were observed in infants and children exposed to As since early childhood (WHO, 2021b). THQ of Cd were all <1.00, as shown in Table 3, indicating the low chronic risks associated with a diet based on commercial baby foods only. HI values based on LB, MB, and UB levels were all below 1.00, suggesting the low chronic risks associated with As and Cd exposure by infants ≤12 months old through baby foods in Saudi Arabia.

EFSA released a report investigating the contribution of Cd to total intake of European infants (under 12 months old) by the consumption of multiple food items, among which stage 1 infant formula accounted for the highest contribution of 20.0%, followed by 9.20% and 1.60% of cereal‐based food and stage 2 formula, respectively (European Food Safety Authority, 2012). In comparison with the previous findings of EFSA, our results of stage 1 infant formula intake indicates higher exposure to Cd, but less exposure to baby foods for 7–12 months infants. Recent literature in Egypt showed lower intake levels of Cd through commercial infant formula (8.67%), while consuming follow‐on formula contributed to higher exposure than results obtained in this study (7.22%) (Trier et al., 2010; WHO, 2021d). Similar results for all infant age groups were observed in the United States (0.00 μg/kg bw/day), Poland (0.05–0.76 μg/kg bw/day), Turkey (0.03 μg/kg bw/day), China (0.01 μg/kg bw/day), and Italy (0.02 μg/kg bw/day) (Bargellini et al., 2018; Başaran, 2022; Gardener et al., 2019; Mania et al., 2015; Su et al., 2020). Similar to As, the presence of Cd, even in scarce amounts, in infant food products is concerning. This is attributed to Cd extreme toxicity and carcinogenicity on humans, especially if children were exposed to Cd during early childhood (WHO, 2021d). Upon in utero, early childhood, and breastfeeding exposure and accumulation, Cd causes developmental toxicity such as decreased growth rate, malformations, low weight at birth, high mortality rate, and chronic diseases in future (Almutairi et al., 2021).

Exclusive breastfeeding for infants under 6 months is ideal, due to enriched nutrients and antibodies content in breast milk (Alshebly & Sobaih, 2016; Ministry of Health, 2021b). In Saudi Arabia, less than 40.0% of mothers practiced breastfeeding for the first 6 months, while the remaining percentage prefer formula milk for infant feeding (Alshebly & Sobaih, 2016). Such facts are due to various causes including lack of breast milk, convenience of formula milk, and special medical cases of infants (e.g., preterm infants) that needs specific type of formula milk (O'Connor, 2009). Assuming that more than 50.0% of infants in Saudi Arabia are fully fed on formula milk, the exposure to As and Cd might be higher than anticipated in this study for more than half of Saudi infant population. Such matter is concerning and awareness of the importance of breastfeeding in the infant's first 6 months should be exercised nationwide.

For infants older than 6 months, many other sources can be associated with increased heavy metals intake. Although none of our findings exceeded the RfD of As or PTWI of Cd, the intake levels may exceed the reference values in some cases and increase the risks on infants. For instance, the use of As‐contaminated tap water to prepare and clean feeding bottles, plates, and spoons may play a part in increasing the intake of As. As previously stated, As and Cd mainly reside and accumulate in cereals and grains, particularly rice. According to the American Academy of Family Physician (AAFP), rice starch could be used in formula attributing to its protein abundancy, hypoallergenic and antireflux potential compared to the standard or soy‐based formula (O'Connor, 2009). In addition, rice‐based meals, teethers, and snacks are often consumed more than other baby food products (Gu et al., 2020). As stated by the Ministry of Health (MOH) in Saudi Arabia, the common daily diet consists mainly of rice and grains; therefore, an infant exposure to higher As levels through daily meals may occur in the early stages of tasting solid foods (Ministry of Health, 2021c). Moreover, this type of phenomena is rapidly growing with increasing incidents of celiac disease (CD) and wheat intolerance in Saudi Arabia among infants, toddlers, and schoolaged children (Saadah & ALNosani, 2020). Hence, providing a balanced diet based on fruits, vegetables, cereals, and grains complementary foods suitable for infants under 12 months may aids in lowering As and Cd exposure.

Based on the obtained data, the MOE values were lower than 10.0 and greater than 1.00, indicating the risk is likely to be low, however, taking an action to lower the contents of Pb in food products for infants is essential. The inclusion of relatively low quantification limits was useful to examine Pb contents at very low levels. Our results were comparable to the German Federal Institute for Risk assessment (BfR) opinion which concluded that the consumption of foods by infants resulted in an exposure of Pb below the BMDL_01_ of 0.50 μg/kg bw/day (World Health Organization, 2016). The foods selected by BfR for the assessment included infant formula, ready‐to‐eat foods, and processed cereal‐based meals tested in the monitoring plan of 2015 (World Health Organization, 2016). Given the sensitivity of infants to Pb, food producers should be directed to reduce the levels of Pb that could be produced during the production of baby foods and infant formula. Similarly, our findings are parallel to other research studies conducted in Europe (IARC, 2021; Srivastava et al., 2017). Due to its rapid absorption and accumulation in infants and children, Pb is a matter of concern when consumed during early life stages (IARC, 2021). It was found that the risk of Pb exposure is low in breast‐fed infants, indicating that commercial infant products might be the dominant contributor to Pb exposure in infants (IARC, 2021).

This study is the first to conduct risk assessment on baby foods consumed by infants under 12 months in Saudi Arabia. Moreover, it can provide a research roadmap for future studies for further investigation. However, there is a lack of specific consumption data for Saudi infants and young children consuming the infant formula of both stages and cereal‐based meals and biscuits. Furthermore, the included baby food samples in the NFMP were restricted to undiluted infant foods (formula and cereals) and ready to eat baby biscuits available at Saudi markets, while composite foods prepared at home for infants aged 7–12 months were not considered in this assessment. Therefore, the actual exposure to heavy metals might be at even lower levels than the results obtained in this study. Additionally, including other baby products that could affect the total intake such as homemade foods, ready‐to‐eat purees, gruels, baby juices, and teething crackers is highly encouraged for future research conducted through NFMP or other research groups and programs. Assessing the accurate dietary intakes of toxic elements by infants is challenging, as there are many factors that could affect their daily intake. At the first year of life, infants commonly have feeding problems. For instance, they usually have impaired gastrointestinal function or allergy reactions to certain foods. The effects that these factors may have on the total dietary intake of infants were not considered in this study. Essentially, the consumption data used to assess the dietary exposure does not essentially reflect the actual intake of infants.

## 5 CONCLUSION

Based on the occurrence data obtained from the NFMP of 2020, the mean concentrations of As and Cd in stage 1 and stage 2 infant formula, cereal‐based foods, and biscuits samples were in accordance with the maximum limits set by SFDA. When assessing the dietary intake of arsenic and cadmium in infants, the exposure levels were below the corresponding RfD values, as well as the levels of THQ and HI (<1.00). This indicates that the occurrence of As and Cd in the examined food groups pose low chronic health risks on infants in Saudi Arabia. Additionally, MOE for Pb revealed that the related health risks are likely to be low with prolonged exposure in infants, however, not in levels that can be neglected. In light of such evidence, the exposure of As, Cd, and Pb in the investigated baby food samples is likely to pose low chronic health concerns. Many limitations were observed including the infant formula and cereal meals samples used in this assessment were tested in dry form, as well as the lack of consumption data for Saudi children under the age of 1 year old consuming infant formula of both stages, cereal‐based meals and biscuits, and finally, the absence of toxic elements exposure evaluation in light of feeding on other homemade baby food and consuming breast milk alongside the tested baby foods in this study. Further research studies are a necessity to examine heavy metals in more diverse food items for infants and young children (e.g., fruits and vegetables porridges), evaluate other toxic elements or contaminants (e.g., mercury and Ochratoxin A), and include other age groups (e.g., 1–3 year old children). Finally, this study might assist in revising the current maximum limits of Pb, among many other heavy metals, in baby foods. This step could tremendously help in reducing the associated probable risks of heavy metals on infants.

## AUTHOR CONTRIBUTIONS

**Najla S. Alharbi:** Data curation (lead); formal analysis (lead); methodology (lead); project administration (equal); visualization (lead); writing – original draft (lead); writing – review and editing (equal). **Rawdah M. Akamsiei:** Data curation (supporting); formal analysis (supporting); investigation (supporting); methodology (equal); writing – original draft (equal); writing – review and editing (equal). **Lama A. Almaiman:** Data curation (supporting); writing – review and editing (supporting). **Mostafa A. AL‐Samti:** Formal analysis (equal); methodology (equal); resources (lead); validation (lead); writing – original draft (supporting). **Hamad S. Al‐Mutairi:** Formal analysis (equal); methodology (equal); resources (lead); validation (lead); writing – original draft (supporting). **Bandar S. Al‐owais:** Data curation (supporting). **Majid M. AlKhalaf:** Writing – review and editing (equal). **Mohammed A. Bineid:** Conceptualization (lead); methodology (equal); project administration (lead); writing – review and editing (lead).

## FUNDING INFORMATION

The authors thank the Saudi Food and Drug Authority (SFDA) for financial support.

## CONFLICT OF INTEREST STATEMENT

The authors of this study claim no conflicts of interest.

## ETHICS STATEMENT

Ethical approval is not applicable to this study.

## DISCLAIMER

The views expressed in this article are those of the authors and do not necessarily reflect those of the Saudi Food and Drug Authority (SFDA) or its stakeholders. Guaranteeing the accuracy and the validity of the data is a sole responsibility of the research team. The authors are not aware of any affiliation, membership, funding, or financial holding that might be perceived as affecting the objectivity of this study.

## Data Availability

The data that support the findings of this study are available on request from the corresponding author. The data are not publicly available due to privacy or ethical restrictions.
